# What matters to patients with cancer receiving home care at the end of life? A qualitative study comparing patients’ and healthcare professionals’ views

**DOI:** 10.1080/17482631.2025.2517358

**Published:** 2025-06-11

**Authors:** Lisbeth Thoresen, Eline Aas, Nikki McCaffrey, Lidia Engel, Nina Løkkevik, Yvonne Anne Michel, Gudrun Maria Waaler Bjørnelv

**Affiliations:** a Department for Interdisciplinary Health Science, Faculty of Medicine, University of Oslo, Oslo, Norway; b Department of Health Management and Health Economics, University of Oslo, Oslo, Norway; c Division for Health Services, Norwegian Institute of Public Health, Oslo, Norway; d Deakin Health Economics, School of Health & Social Development, Institute for Health Transformation, Faculty of Health, Deakin University, Burwood, Australia; e Health Economics Group, School of Public Health and Preventive Medicine, Monash University, Melbourne, Australia; f Research and Development Department, Moss, Norway; g Faculty of Social Sciences, University of Applied Sciences Zittau/Görlitz, Görlitz, Germany; h Department of Public Health and Nursing, Norwegian University of Science and Technology, Trondheim, Norway

**Keywords:** Cancer, end-of-life care, home care, patients’ experience, healthcare professional, qualitative

## Abstract

**Purpose:**

To improve care for patients in the last phase of life, healthcare professionals (HCPs) need to understand what matters to them in terms of care and follow-up. Therefore, in our study, we investigated how patients with cancer in Norway who are receiving home care perceive their situations and what matters to them at the end of life and compared it with HCPs’ views on what matters to such patients.

**Methods:**

Following a qualitative design, we conducted in-depth interviews with eight patients with late-stage cancer living at home and four focus group interviews with 21 hCPs, mainly nurses. Patients and HCPs were recruited from two municipalities in Southeast and mid-Norway. The study period lasted from December 2020 to October 2022. We performed reflexive, thematic analyses of both data sets.

**Results:**

The views of all eight patients and all 21 hCPs were interpreted according to five themes: cancer impacts all aspects of life, navigating the healthcare system, living with dying, the paramount importance of relationships, and dying at home.

**Conclusion:**

Entering the end of life, patients feel exhausted, lonely, and abandoned. They lack confidence in HCPs and the healthcare system. HCPs characterized plans and predictability as being important during end-of-life care, whereas patients were often hesitant to talk about and plan for the last phase of life.

## Introduction

In Norway, out of a population of 5.5 million, approximately 300,000 people will be diagnosed with cancer during their lifespan. In 2023, the four most common types of cancer in Norway were prostate, breast, lung, and colorectal cancer (Cancer Registry of Norway, 2024). However, due to advances in cancer screening, early detection, and medical treatment, people increasingly survive cancer (Shapiro & Longo, 2018). In Norway, three out of four people with cancer are alive five years after diagnosis, and increasingly more patients fully recover (Cancer Registry of Norway, 2023). Even so, cancer has been the leading cause of death in Norway since 2017 and accounted for 25% of all deaths in the country (11,451 out of 45,774) in 2022 (Cancer Registry of Norway, 2023). However, little is known about the needs of patients when cancer treatment ends and death is imminent, either at home or in a healthcare facility (Helse- ogomsorgsdepartementet, 2020; Meneses-Echavez et al., 2021; Michel et al., 2024).

### Healthcare, cancer care, and palliative care in Norway

In Norway, healthcare services are primarily funded by taxes and generally free at the point of access, although some services require out-of-pocket co-payments up to a certain amount. Primary care, home care, and community-based care, including physiotherapy, nursing care, practical assistance, short- and long-term institutionalized care, and general practitioners (GPs), are provided at the municipal level. GPs are responsible for assessing, diagnosing, treating, and following up with their patients and serve as gatekeepers to secondary healthcare services. Secondary and tertiary care, primarily administered in hospitals, are financed and provided by four state-owned regional health authorities (Aas et al., 2021; Tikkanen et al., 2022).

In 2015, standardized cancer patient pathways were implemented in healthcare services in Norway with the aims of reducing and standardizing wait times and improving predictability for patients—for example, when they should receive treatments such as surgery or chemotherapy (Nilssen et al., 2020; Solbjør et al., 2021). The standardized pathway begins when a person suspected of having cancer symptoms is referred to a hospital, often by their GP, and ends when cancer treatment commences. After patients receive information about the pathway and tests needed to determine whether their symptoms are caused by cancer, they can contact a cancer pathway coordinator at the hospital if they have any questions concerning practical matters related to their assessment and appointments, among other topics (Helsedirektoratet, u.å.). Once cancer treatment is completed, patients are usually followed up for the next 3–5 years. Although GPs once played minor roles in cancer treatment in Norway, today’s GPs may participate in aspects of oncological treatment, clinical follow-up, and palliative care. In rural areas of Norway, owing to long distances between patients’ residences and hospitals, the GP’s involvement is particularly important (Holtedahl et al., 2018). Cancer coordinators in municipalities support patients with cancer and their next of kin coordinating relevant health care services and possibly by acting as a contact between them and hospital services. The cancer coordinators are an important part of cancer care in Norway, including at the end of life.

Current guidelines in Norway emphasize the integration of cancer and palliative care and close collaboration between hospitals and municipalities (Brenne et al., 2020; Helse og omsorgsdepartementet, 2017; Kaasa et al., 2018). Palliative care is provided in hospitals, outpatient clinics, and nursing homes and as part of home care. Although pathways exclusively for palliative care have been proposed, they have not yet been established (Helse og omsorgsdepartementet, 2017). Norway’s policy on palliative healthcare advocates an open awareness of dying and death, holistic approaches, and patients’ involvement, including the freedom to choose where to die (Helse- ogomsorgsdepartementet, 2020). Of Norway’s general population, 53% of individuals prefer to die at home (Fransiskushjelpen, 2021). However, in 2023, only 14.5% of people did die at home, a statistic that includes both planned and unexpected deaths (Folkehelseinstituttet, 2024). Increasingly, emphasize is placed on the number of days that patients spend at home instead of whether they die at home (Helse- og omsorgsdepartementet). Irrespective of diagnosis and sex, younger and married patients tend to spend more days at home (Bjørnelv et al., 2024).

### Home care for patients with cancer approaching the end of life: needs and preferences

In the context of cancer, end-of-life is defined as the period when curative cancer treatment has been stopped and the patient is likely to die within the year (Scobie & Hutchings, 2022). Palliative care, another term used in this article, is not synonymous with end-of-life care but is an approach “to prevent, treat and reduce the patient’s symptoms and suffering caused by treatment toxicity and tumour burden and to preserve and improve psychological and social well-being” (Kaasa et al., 2018, p. 6).

When patients with cancer approach the last phase of life, the prevalence of physical symptoms including pain, loss of appetite, shortness of breath, and health instability often increases (Seow, Guthrie, et al., 2021), along with psychosocial symptoms such as social decline, cognitive impairment, and depression (Seow, Stevens, et al., 2021). Thus, comprehensive palliative care needs to address patients’ existential, social, and spiritual needs and worries as well as physical symptoms (Khan et al., 2014; McCaffrey et al., 2024; McCaffrey et al., 2009), and the systematic registration of symptoms is recommended (Vigstad et al., 2018). According to the Norwegian Cancer Strategy, patients with cancer should have access to information on their diagnosis, treatment, side effects that may emerge later, and where they can seek advice, support, and treatment (Helse- og omsorgsdepartementet, 2018). To provide care that aligns with what patients with cancer prefer, advance care planning is described as the cornerstone of end-of-life care (Driller et al., 2022; Khan et al., 2014). Many patients with cancer prefer to receive palliative and end-of-life care at home (Lersveen & Devik, 2021; Peoples et al., 2020), especially when approaching the late palliative phase (Nysæter et al., 2022). In 2023, 14% of home deaths in Norway were related to cancer (Folkehelseinstituttet, 2024). However, it is unclear whether these deaths were planned (Kjellstadli et al., 2018). According to Hov et al. (2021), 60 palliative care patients in Norway who responded to a questionnaire on their experiences felt safe or fairly safe while receiving home care, whereas another study conducted in Norway characterized the help received from healthcare services as being fragmented and unable to meet patients’ and/or families’ needs (Fjose et al., 2018). Patients receiving home care for late-stage cancer have usually been able to adjust their everyday lives to maintain a good quality of life (Peoples et al., 2020).

### Research questions

In Norway, guidelines for palliative care (Helsedirektoratet, 2019) and cancer care pathways describe how cancer care and end-of-life care should be organized. However, little is known about how end-of-life care is provided and experienced by the patients themselves. Therefore, in our study, we investigated what matters to patients with cancer in Norway, living in their private homes (i.e., not institutions) and facing death. In the process, we aimed to examine whether healthcare professionals (HCPs) in the municipalities, who often have the primary responsibility for those patients, share the patients’ views in terms of what matters to them. Accordingly, we formulated two research questions:

- How do patients with cancer who are receiving home care and approaching the end of life perceive and experience their situations, and what matters to them?
- How do HCPs perceive and reflect on the home care received by patients with cancer and what matters to them?

## Methods

### Context and research team

Our study was part of a larger interdisciplinary research project titled “Securing High Quality Care for Patients with Cancer at Their End-of-Life” (“the SAFE Study”) conducted in the Department of Health Management and Health Economics at the University of Oslo. The chief aim of the SAFE Study is to increase knowledge about the current care pathways of patients with cancer at the end of life regarding their living situation, perceived quality of life, and use of healthcare services. We also aimed to contextualize our registry-based quantitative analyses using a qualitative methodology (Bjørnelv et al., 2022; Bjørnelv et al., 2024; Michel et al., 2024).

To achieve research synergies as an interdisciplinary group of health economists and nurses, we had to work together, which required an open mind and interest (Trussell et al., 2017). For example, we simulated qualitative individual interviews to better understand the design of the method and the ethical challenges related to conducting interviews with patients approaching death.

### Design, recruitment, and participants

To deepen our understanding of what matters to patients with cancer who are receiving home care and approaching the end of life, we adopted a qualitative design. Within this design, our methods were chosen to gain a better understanding of the experiences and views of patients with cancer and compare their views with the views of HCPs, all to enhance patient-centred care and draw attention to gaps between patients’ situations and healthcare staff’s perceptions of those situations (Lindsay, 2019; Sublette et al., 2017).

The study included patients with cancer who were (1) >18 years old, (2) living at home, (3) at the end of their lives, and (4) informed of their shortened life expectancy. Patients were excluded if they had a serious mental illness or if they were in the terminal phase of their illness (i.e., final days or hours). We planned approximately 15 individual interviews with patients and three to four focus group (FG) interviews with HCPs from two municipalities in Southeast and mid-Norway. Nurses and other healthcare staff with at least one year of experience caring for patients with cancer in the palliative phase were eligible to participate. Cancer coordinators from the two municipalities were in contact with patients with cancer who were staying at home. The cancer coordinators identified 14 eligible individuals and sent them written invitations to participate in interviews. Patients were asked to respond to the cancer coordinator within a week. The sampling strategy for HCPs was purposive, and they were recruited to participate by leaders in the health care services in the two municipalities. The only criterion for participation was at least one year of experience caring for patients with cancer in the palliative phase. There were no other eligibility criteria. The recruitment and interview process lasted from December 2020 to October 2022. Appropriate precautions were taken for the face-to-face interviews with the patients, in line with the existing COVID-19 protocols in existence at the time.

### Data and analysis

Individual interviews with patients were conducted by a nurse and a health economist, took place in the private homes of patients with cancer, and lasted 20–60 minutes, with 60 minutes being the maximum to not exhaust the patients. Along with the individual interviews, we also conducted FG interviews with HCPs as virtual meetings led by the first author which lasted approximately 40 minutes each. The interview guides for the individual and FG interviews addressed similar issues but were formulated differently because more sensitive formulations were necessary for interviews with patients. The patients were asked at the beginning of their interviews to describe their everyday lives, what kinds of formal and informal support they were receiving, and what mattered to them at their stage of the disease. The HCPs, by contrast, were asked to describe how home care for patients with cancer was organized and administered, and among other issues, what they thought mattered to patients with cancer at the end of life. Appendix 1 presents the interview guides. All interviews were audio-recorded and transcribed verbatim.

Shortly after the individual and FG interviews were completed, four Norwegian members of the research team discussed what was particularly interesting, surprising, and touching. The results informed our preliminary data analysis (Grbich, 2013). Subsequently, the same four team members read the interview transcripts and discussed their thoughts regarding the content. Familiarizing oneself with the data is the first step in reflexive thematic analysis (Braun & Clarke, 2022), which is often used when comparing groups in health-oriented research (Lindsay, 2019). The first author coded parts of the transcripts related to the research questions, which were mostly summative, and subsequently generated and refined the themes identified therein. These themes have been discussed and agreed upon by other authors. In analysing and writing our results, we aimed to foster sensitivity to what the interviews revealed while following a questioning, open-minded approach (Braun & Clarke, 2022). Patients’ names are pseudonyms, and when referring to HCPs, we labelled quotations with a capital letter and a number that corresponds to the FG and municipality, respectively.

Research rigour was ensured by providing adequate background information for this study, crafting a clear research question and aim, and describing the method and research process in detail with transparency. Further, a broad, interdisciplinary research group discussed every stage of the study and participated in analysing and writing up the results. An important aspect was reflecting on the researcher’s role and potential biases throughout the research process, particularly the analysis.

### Ethics

Sharing experiences about serious illness and facing death are sensitive topics for many individuals, including the participants in our study, and require the interviewer to be attentive and sensitive. This study was conducted in accordance with the principles of the Declaration of Helsinki. All participants signed an informed consent form, and the data were stored on a secure research platform at the University of Oslo. The study was approved by the Regional Ethics Committee (No. 112008) and by SIKT (i.e., for the protection of privacy; No. 180225).

## Results

Transcripts from eight individual interviews with patients with cancer from both municipalities (i.e., Municipalities A and B) and four FG interviews from both municipalities were available for data analysis. All patients interviewed were over 65 years of age and had last-stage cancer (Table I). Meanwhile, the FG participants consisted of 21 women, primarily nurses, whose ages ranged widely and some of them had a master’s degree in cancer care or palliative care.

**Table I.** Participating patients’ characteristics.

Name	Age	Marital status, children, and social connections	Cancer history and stage of treatment	Services and healthcare professionals
Ingrid	90	Widow; only child is dead	Newly diagnosed	Daily home care, cancer coordinator, and GP
Eric	60	Married; has children	Cancer for years; end of curative treatment	GP, hospital, cancer coordinator, and physiotherapist
Peter	65	Married; has children and grandchildren	Cancer for years; end of curative treatment	Cancer coordinator
Anna	85	Married; has children, grandchildren, friends, and neighbours	Cancer for 2 years; end of curative treatment	Daily home care, cancer coordinator, and hospital doctor
Paula	75	Married; has children, grandchildren, friends, and neighbours	Diagnosed with cancer three times; end of curative treatment	Cancer coordinator
Jan	75	Newly widowed; little contact with children	Cancer for 2 years; end of curative treatment	Home care once per week, hospital, and cancer coordinator
Sissel	70	Unmarried; little contact with children	Cancer for years; end of curative treatment	Daily home care and physiotherapist
Jens	70	Married; family in the neighbourhood but misses friends	Cancer for years, end of curative treatment	Cancer coordinator, hospital, and outpatient care

Table I provides an overview of the participants included in this study. Names are pseudonyms, and ages have been rounded to the nearest 5-year age group.

This analysis revealed five key themes. In this article, the findings from the interviews with patient are first presented, followed by an analysis of the FG interviews. The approach prioritizes patients’ views and experiences before comparing them with how HCPs understand the home care received by patients with cancer and their needs. To illustrate our claims, we use quotations from interviews (Braun & Clarke, 2022).

### Cancer impacts all aspects of life

In individual interviews, all seven patients excluding Ingrid, who was newly diagnosed, were experienced patients with cancer. They had learned the hard way how living with a serious illness can take over one’s life, body, and relationships. They reported how years of intensive cancer treatment, a high symptom burden, and emotional, social, and existential challenges left marks on them. During the interviews, it was evident that their histories with the disease influenced their reflections on their current situations and their thoughts about the future. To understand patients’ daily lives and their outlook on the future, it is important to recognize how cancer has impacted their lives in various ways for years.

In the FG interviews, HCPs reported that most patients with cancer whom they had met in the municipal setting had long been in the healthcare system, primarily in hospitals for cancer treatment, before receiving home care in the municipalities. One cancer coordinator described how “some pathways may last for months and years before the palliative phase begins. People are already worn out when they enter the challenging last phase” (S2). Even if healthcare professionals (HCPs) can never fully understand the personal experiences of patients, they are aware of the often long-lasting burden faced by cancer patients.

### Navigating the healthcare system

According to the individual interviews, cancer coordinators represent a lifeline and safety net for patients: “She [the cancer coordinator] can help us when we need it; it’s someone to ask for help from” (Peter). In Jan’s case, the cancer coordinator visited regularly and was described as always being accessible: “I have the phone number if I need something”. Conversely, patients indicated that their interactions with different healthcare personnel and healthcare departments over the years had revealed a lack of cooperation and communication both among the HCPs and with patients. These experiences were not only related to the past but continued to be part of their daily lives as they approached the end of life. Paula, for instance, had received three different cancer diagnoses and three different hospital departments following up on her symptoms. According to her, different departments did not collaborate, and she described the lack of holistic care in her situation. She was not the only patient with a complex history of illness. In addition to cancer diagnoses, including metastatic cancer, patients also lived with other long-term chronic conditions and, as in Eric’s case, had to “run between doctors and specialists for the last four years. They look at you and check you off as if you’re finished. At that point, it seems like they’re not responsible [for you] anymore, so no one can blame them for anything”. Eric asked rhetorically which HCP has an updated overview of his medication, and his answer was that “It’s only me who knows”. Eric knew that as part of the cancer pathways, he should have one contact person, one who is the “traffic director”, so to speak. However, according to Eric, “No one takes responsibility”. The lack of a traffic director also emerged when other patients discussed their former and present situations, especially concerning the lack of information available and their experiences with being rejected by healthcare services. As Sissel described it:

From the beginning, I was told that the [hospital] departments cooperated, but they did not. They work separately, and the body is put into pieces. They did not talk with each other. I am ill, and I think that I could have been spared much of my worries and anxiety. I get worn out by fighting. I’ll call it a war. Like tomorrow, I’ll have a chemo, but after that? I know that they are busy, but it has been like that for years. No one believes what I have been through.

For the patients, experiences of not being informed and involved but instead being overlooked were followed by a sense of loneliness. Paula described how she felt abandoned during the summer due to not having any contact with the hospital, and Anna summarized her communication with the healthcare services as follows: “I feel that everyone has left me, that there’s no one who can help me”.

In the FG interviews, except for the cancer coordinators who may have had some contact with patients during the period of active treatment at the hospital, the HCPs’ encounters with patients with cancer in the municipalities primarily began when the patients entered the palliative phase—for many, when they were approaching the end of life. In all four FG interviews, HCPs characterized the cancer coordinator as significant in supporting, guiding, and coordinating healthcare services for patients with cancer. However, in one of the municipalities, fewer than half of all known patients with cancer were in contact with the coordinator, and overall, all four groups described that not all patients with cancer were identified and got the help they needed: “There are many [patients with cancer] whom we don’t know about” (M1).

The transition from active curative treatment in hospitals to palliative and end-of life care in municipalities was described in FG interviews as challenging for patients. Such difficulty arose because they had to interact with many new HCPs and understand the organization of the municipality’s health services. They also needed to learn who was responsible for what, including the roles of home care services, cancer coordinators, palliative care units in nursing homes, GPs, and hospitals. One cancer coordinator described the cooperation between the different professionals as “the ongoing dynamic process around patients with cancer” (M1). In the same FG, a home care nurse rephrased the “dynamic process” when reminded about all the different people that patients with cancer had to deal with: “There are too many of us. There are new faces all the time” (M1). The HCPs also emphasized how rapid changes in a patient’s situation could be frequent: “Situations arise, and suddenly the next of kin become resigned, and the patient cannot stand it any longer” (M2). These changes call for flexibility and competent professionals, and the FG interviews highlighted the need for improved collaboration between all HCPs and systems involved: “A palliative care pathway could have helped, especially to respond to patients’ preferences” (M2).

This result shows that patients and HCPs agreed on the significance of the cancer coordinator in coordinating health services and supporting the patient. Both groups pointed to the need for better support and collaboration among HCPs involved in patient care.

### Living with dying

Curative treatment had ended for all patients, and some had lived longer than expected. At the time of the interviews, our impression was that the patients were aware that they were facing death; however, how they reflected that knowledge varied. Eric, for instance, described having accepted the situation and claimed he took things as they came. Some patients had even finalized their affairs and prepared their funerals. Jan was stoic about the future: “Everyone goes through it [death], and I do not think that it is something to worry much about. But nobody looks forward to the agony of death. I do not know how long it takes”. By contrast, there was also resistance and avoidance among the patients, as Peter exemplified:

We [Peter and his wife] have touched upon it [the future]. But we take things as they come; we live here and now. Contact with the cancer coordinator can wait. … We can wait until the condition worsens; we do not have to go any further now. It is not necessary to dig into it; I do not have to seek out the shit. I try to see the best in things. … I can wait a bit.

Anna added that she had been encouraged by the cancer coordinator to accept the reality of her situation: “I can’t accept it [death] yet. I have not reached that point”.

In the FG interviews, Peter’s approach of taking things as they come resonated with the HCPs’ descriptions of how some patients with cancer in the municipalities did not want to make plans:

Some patients do not want to contact cancer coordinators because they want to wait, and we get a bit worried because it is time for them to make plans, but they do not want to. Suddenly, they become very ill and soon die, and the last phase turns out to be what none of us wanted. (M1)

Even if HCPs listened to patients’ wishes, asked them what they wanted, and expressed “walking in step with the patients” as being an ideal (M1), when it came to planning the future, they wanted to start early: “It is very important that we stay ahead of the situation. We cannot wait. That’s the alpha and omega: thinking ahead” (S2). Another HCP added, “Predictability, particularly when the situation worsens, is crucial” (M2). HCPs also sometimes worried that patients were unaware that they were indeed dying or that their time was limited and what the final phase might be like: “The situation demands that they [the family and patient] talk together, and we can help them to talk—perhaps a family conversation to clarify expectations” (S1).

We have a kind of advance care planning—it’s not very formalised—in which we clarify who knows what. Who’s in charge? The palliative care team or the GP? Is it possible for the patient to be admitted to the nursing home? Who is the responsible nurse in home care? Knowing the name is important to the patient in order to know whom to contact. (S1)

This result indicates that perspectives may differ between patients and HCPs as patients approach the end of life. While HCPs often prefer to make plans for this stage, patients may not be ready to engage in such discussions.

### Relationships are most important

In individual interviews, when asked about what was important in their current situations, all eight patients highlighted the significance of different relationships. Being part of those relationships was meaningful and important to them in the last phase of life, and they often expressed a desire to stay at home for as long as possible. To Paula, staying at home meant “having easy access to the beauty surrounding me: the garden, birds, flowers” and perhaps also what Eric indicated: “You have to … use your energy on the good things”. Among Peter’s “good things”, his family was the foremost:

I’m happy to be alive. I can do something other than simply stay on the sofa. I can see my grandchildren. Looking back, I am happy with my life. I wish that I could have lived longer to see my grandchildren grow, but they will remember me. I do not have pain. [It’s also important] To be with my wife. Quality of life means that we two are – that I am not alone. Relationships are the most important thing.

Children and grandchildren were mentioned frequently, as were friends and neighbours, but so was continuing to be part of different groups with a shared interest in sport, dance, or sailing, among other things. Meeting with those peers was meaningful because they had something besides illness in common, and it afforded distraction and social connection. Jan said that he was lucky to meet with friends at the local café nearly every day and described how it had helped him to avoid feelings of loneliness.

However, there were also differences in whether and how cancer was discussed between the patients and their family members and friends, and some wanted to spare their families with bad news. Doing so meant that patients could be quite alone in dealing with their worries and pain, as Sissel described: “I wear masks: one at home and one when I’m out among others”. If relationships were lacking or strained, it contributed to an already difficult, sad, and often passive life situation. A couple of patients could go for walks and/or drive and meet with others, but pain and other troublesome symptoms, problems with balance, and fatigue restricted everyday life for most patients. Time was spent resting, sleeping, watching TV, and trying to eat something and was described as passive time, as if one were captive. Many patients shared Anna’s experience of how days had become similar and terribly bored. While they lived in “slow motion”, everyone else continued their hectic lives, particularly children and grandchildren, as the following quotation shows: “You know, everyone’s busy, and they [my children] don’t find it particularly meaningful to visit me” (Jan). Jens added that he had lost many friends after being diagnosed with cancer, and he had also heard similar stories from others:

It hurts, because you are not leprous or contagious. … and that woman, she described how she had lost nearly all her friends. None of them visited, like they used to. … When you get cancer, meeting with friends and having someone to talk to are essential.

Meanwhile, in FG interviews, a recurring idea concerning what matters to patients in the final stages of cancer was that such patients are diverse and so are their needs and desires: “Some have many relatives and a close network. Others are on their own, and there may be many challenges for the patient. Different aspects impact the situation” (S1). Another HCP added, “There are no definitive answers to those questions” (S2). However, HCPs also confirmed that spending time at home with close family members was generally important to patients with cancer in the last phase of life, as was improved quality of life. “Keeping up a sense of normalcy in everyday life and living until you die” (M2) were part of that notion and something that HCPs supported as much as possible.

Unlike the patients, no HCPs mentioned friends, neighbours, or memberships in clubs as significant for maintaining a social life. HCPs spoke more about predictable pathways and plans, as mentioned earlier, but also highlighted cooperation, continuity, and the availability of staff as things that mattered to patients. Additionally significant was competent palliative care: “Soothing the pain is the most important thing. Yes, [soothing] pain and sickness. Anxiety. Knowing how to continue, how to take the next step. … If the HCPs are assured, then the patient will feel safe” (S2). Some HCPs highlighted more existential aspects, including the losses and sorrows related to becoming seriously ill and facing death: “Often, there’s loneliness: an inner feeling that you are on your own, that you will lose your life and say goodbye to people close to you” (M1). Another existential aspect raised was the patients’ need to “experience that you remain yourself, as long as possible, and have some kind of control, have something you want … to be taken seriously” (M2). Related to those aspects was being attentive to the patient’s preferences, especially when their preferences change, as they often do, either in the course of the disease or “towards the end-of-life” when “they suddenly change” (M2).

What matters to patients with cancer in this study resonates in many ways with the perspectives of HCPs. Both groups agreed on the importance of close relationships; however, patients also emphasized the significance of broader social connections to improve their everyday lives while living with a serious illness.

One preference that might change was the preferred place of death.

### Dying at home

In individual interviews, patients mentioned the place of death, and some wanted to spend the remaining time at home and die at home, if possible. However, those who lived with their spouses discussed the pressure on their loved ones, which could be too much: “But then there’s my wife. There is a limit to what she can bear” (Peter). Others, including Eric, contemplated the hospital as being the best place to die: “I don’t know. In some ways, it may be safer to be in the hospital if you have severe symptoms, and they [HCPs] can control your pain. You cannot expect your family to become HCPs in 24 hours. But that will soon be the case. … If I were to die at home? I cannot relate to that”. Jan was clear about what he wanted: “When I get weaker, I want to move into a nursing home”. According to Jan, the cancer coordinator had stated that they would try to grant his wish but that they could not make any promises. Jan was unsatisfied with the answer. To him, the wording was too non-committal. He was afraid to be left at home without help when his situation worsened, and he wanted assurance that he would be transferred.

According to HCPs in FG interviews, seriously ill patients with cancer are not always informed about the possibility of dying at home, while others are reluctant to talk about or plan for the end of life and where they wanted to die: “When I’ve asked [patients about that], I’ve sometimes been roundly rejected” (M1). It seems that planning for one’s impending death was an admission that you were going to die, and, as mentioned, not all patients were ready for or had accepted that reality:

They did not want contact with the cancer coordinator. They will wait. We are aware that it is time to plan the last phase, but they do not want to. It is quite frustrating for us because we see that they are struggling, but they do not dare to take the step to start planning. And then it gets hasty. Suddenly they become ill and die within one week. And nothing turned out like we wanted it. (M1)

An important aspect of place of death, one that both patients and HCPs underscored, is that patients do not want to be a burden to their families and thus would rather die in the nursing home or hospital: “They want to stay at home for as long as possible, but when the situation worsens, they want to go to the palliative care unit at the nursing home in order to relieve their next of kin” (M1). Not necessarily dying at home but spending time at home was mentioned as the most important thing: “I’m happy about this new concept of ‘time spent at home’. But not necessarily at-home deaths. Preferences concerning end of life change suddenly” (M2). However, if a patient wanted to stay at home for as long as possible but wanted to die elsewhere, then diagnosing the patient as dying and admitting them to the nursing home or hospital in time was not always easy to coordinate. The HCPs described how some patients died only a few days, if not hours, after they had been admitted to the nursing home (S2).

The HCPs confirmed that at-home deaths could be challenging in different ways: “Even if home care is supportive, dying at home requires something from the family and the next of kin. It demands a lot of communication and cooperation” (M2). The HCPs described how patients’ homes can be transformed into specialist hospitals, how patients may need care and attention 24/7, and how their families must tackle bodily changes. Some patients were described as vulnerable and insecure towards the end. Considering the challenges of dying at home and caring for someone at home, HCPs emphasized that ending one’s life in a healthcare institution was not a defeat. Even if the home healthcare service wants to support at-home deaths, caring for dying patients at home places tremendous pressure on not only the next of kin but also staff: “It is hard on everyone in the last 2 or 3 weeks. Not only the HCP who is close to the dying patient, but everyone is affected. Perhaps someone else must do your work because the dying patient needs you. It’s teamwork” (S2). However, the experience of being quite busy and stressed should not be visible to the dying patient: “when we visit a dying patient, we pretend that we have all the time in the world” (S1).

As shown by this result, some patients prefer to spend their final time at home and die there, a choice that HCPs agree should be an option. However, both groups express concerns about the burden this decision may place on family members.

## Discussion

We conducted individual interviews with home-based patients with late-stage cancer about their experiences of being seriously ill and receiving healthcare and what mattered to them at the end of life. FG interviews with HCPs somewhat confirmed patients’ perceptions and what was important to them, but there were also differences in patients’ and HCP’s views. What characterized the participating patients in our study was that nearly all had lived with cancer for years; they were older people and were all approaching the last stage of life.

Owing to advances in cancer treatment, more patients with cancer live longer with advanced or metastatic cancer (Boele et al., 2024), and cancer is increasingly understood as a chronic disease (Kaasa et al., 2018). Both participating patients and HCPs described how years of cancer treatment, living with severe symptoms, balancing clinical services and everyday life, and often feeling like a burden to relatives had exhausted patients. Those experiences, as well as information deficits, worries, and uncertainties about the future, have been described in systematic reviews as the unmet needs of people living with advanced-stage cancer (Dunn et al., 2017; Kotronoulas et al., 2017; Moghaddam et al., 2016). The patients’ emotional concerns and needs for supportive care are also highlighted in such reviews, and for that reason, HCPs should inquire about care-related needs to a greater degree (Kotronoulas et al., 2017). Healthcare professionals need to engage more with the needs and concerns of these patients. However, time is a very limited resource when providing home care and will become even more restricted in the future due to a lack of healthcare professionals. Therefore, the establishment of voluntary services to address the emotional and practical needs of seriously ill patients is highlighted in contemporary healthcare policies (Helse og omsorgsdepartmentet, S. m., 2018; omsorgdepartementet, Helse- og, 2023). However, these resources (volunteers and informal care) should be viewed as part of the total resources available to meet patients’ needs and should thus be studied in connection with society’s overall demand for care.

Participating in our study were mostly older adults. We did not plan for that circumstance, as our inclusion criterion was simply being at least 18 years old. However, the patients who were ultimately recruited were all older, likely because people diagnosed with cancer are often older. In Norway, “Half of all people diagnosed with cancer are aged 71 or older, and 90% are over 50” (Cancer Registry of Norway, 2024). Remarkably, none of the patients, HCPs, or members of the research group mentioned older age as a dimension of at-home palliative cancer care. Older age seems to be a nearly invisible aspect of palliative care, and little is known about the quality of life of older patients with cancer (Drageset et al., 2021; Pergolotti et al., 2017; Scotté et al., 2018). Likewise, in a recent study on psychological burdens among 416 patients with cancer, the mean age was 67 years; however, in presenting and discussing the results, the aspect of age was not mentioned (Boele et al., 2024). Nevertheless, there is growing awareness that older people are generally underrepresented or discriminated against in clinical studies on cancer and cancer care (Hurria et al., 2015; Kaasa et al., 2018; Montroni et al., 2022). Such patients may have especially complex social situations, reduced mobility, and increased needs for emotional and practical support (Macmillan Cancer Support, u.å.). Awareness of age-related health issues and taking a geriatric perspective on oncological and palliative treatment may improve care and treatment planning, as well as communication and decision-making processes (Hamaker et al., 2022; Rostoft et al., 2021).

Although the patients in our study were in the last phases of life, how they coped with facing death ranged from stoic acceptance to avoidance for as long as possible. Some wanted to stay where they were at the moment and did not consider the future. For the latter group, there may be tension between them and contemporary ideals of patient involvement and predictable plans in palliative care guidelines and policy development. To improve patients’ participation in palliative care decision-making processes, HCPs should facilitate conversations regarding end-of-life care (Helse og omsorgsdepartementet, 2017; Kaasa et al., 2018; Thoresen & Røberg, 2022). According to Norwegian national recommendations (Helsedirektoratet, 2023), patients with serious and incurable illnesses should be offered advance care planning discussions, which should also involve the municipal health and care services. The GP should participate, and if the patient wishes, their next of kin can be included as well. Through these discussions, the patient’s views and preferences can be understood, and plans can be made accordingly.

The HCPs in our study described initiating conversations with patients and their families about death and, in that way, involving them in planning the end of life. It was important for the HCPs to be prepared for what they knew was coming. However, the HCPs also described encountering seriously ill patients who resisted talking about their impending death and making plans for how to be cared for. That finding seems to counter the fact that patients in our study generally wanted more information and predictability. When it comes to dying and death, some patients become more hesitant and even unwilling to talk, be involved, and make plans. Talking about death and making plans can be understood as admitting that you are dying, which may be too difficult for some. Even if an open awareness towards death and dying may be a contemporary ideal and described as a condition for a good death (Helse- ogomsorgsdepartementet, 2020; Zaman et al., 2021), and even if advance care planning is a tool to improve patients’ involvement, HCPs should also respect that some patients do not want to talk about or plan certain aspects of their healthcare and futures.

All patients in our study lived at home, and five of them lived with their spouses. In Norway’s palliative care policy and guidelines, spending the last phase of life at home is emphasized as an indicator of quality (Helse- ogomsorgsdepartementet, 2020; Helsedirektoratet, 2019). Even if participating patients described all the hours spent at home as being pleasant and meaningful, patients also characterized time spent at home as being quiet, slow, and boring. They additionally described how they could feel lonely and abandoned, as was the case both for ones who lived alone and for ones living with their spouses. Among Norwegians at least 67 years old, 35% live alone (SSB, 2019). Living alone may lead to loneliness and isolation, particularly among older adults (Dybbroe, 2020). Because HCPs in home care today have less time to spend meeting patients’ social needs than in the past, the negative aspects of being on your own may be intensified (Vabø et al., 2022). However, our data also show the strength of “weak ties” (Granovetter, 1973). Close family members were indeed important to the patients, but they also described encounters with more distant others such as neighbours, former colleagues, and members of social clubs, as well as the significance of having something in common with them. These activities could be understood as strengthening the participants’ “identity and bringing joy and a sense of belonging” (Peoples et al., 2020, p. 729), and should thus be recognized to a greater extent by HCPs.

Time spent at home may change when people face death. Bjørnelv et al. (2022) found that patients with cancer during their last 6 months of life on average spent 123 days at home, 24 days in hospital, and 40 days in short- or long-term nursing homes. Those results reflect registry data from 2009 to 2013, and the situation might have changed since healthcare policy began placing even more emphasis on the private home as the place to receive healthcare at the end of life and to die. In our study, HCPs also described how some patients wanted to stay at home for as long as possible but wanted to die elsewhere. The patients did not want to burden their families, and staying at home may also be a question of feeling safe when health conditions worsen (Fjose et al., 2018). When planning end-of-life care, it should be noted that during the final 6 months, patients who live alone spend fewer days at home than ones “with access to informal care” (Bjørnelv et al., 2024).

In closing, we want to spotlight the differences between how people with cancer suspect symptoms quickly become included in standardized cancer patient pathways and the effort put into information and support in the early phase, whereas no such pathway exists in palliative and end-of-life care. Thus, though the pathway at the beginning of cancer seems well-marked, the last phase does not, which leaves patients largely on their own. All patients in our study also described that lack of information and uncertainty when navigating the healthcare system. Being diagnosed with cancer creates fear and anxiety for many patients, often along with disorientation, uncertainty, and senses of loss and a loss of control (Little et al., 2022). Although pathways for cancer treatment may reduce the pressure on patients, the existential experiences may persist throughout treatment and follow-up (Larsen et al., 2022). To improve transitions between different healthcare services and levels and to “ensure continuity and coordination of care in a seamless way”, the need for standardized palliative care pathways is highlighted by the Lancet Oncology Commission on the Integration of Oncology and Palliative Care (Kaasa et al., 2018, p. 6). The experiences that the participants in our study shared with us confirm the need for rethinking future end-of-life care. Perhaps palliative care pathways should be part of the solution.

### Limitations

Our study had limitations. We initially planned to conduct individual in-depth interviews with 15 patients with cancer, inviting 14 to participate. However, only eight patients were ultimately interviewed. Two patients passed away just before their scheduled interviews, two declined, and two became too ill to participate. Cancer coordinators faced challenges in recruiting eligible patients. Some patients struggled to accept their limited life expectancy, making it hard for cancer coordinators to approach them about the study. In some cases, coordinators advised patients to prioritize time with family over research participation. HCPs may have acted as gatekeepers, aiming to protect patients from interview burdens (Kars et al., 2016). While this approach has validity, Bloomer et al. (2018) suggest that seriously ill patients appreciate opportunities to participate in research, as it fosters a sense of value and empowerment. The Covid pandemic strained HCPs in municipalities, possibly reducing patient recruitment priority. Patients may have hesitated to engage in face-to-face interviews due to the pandemic. Similar studies like Nysæter et al. (2022) and Peoples et al. (2020) describe challenges in recruiting late-stage patients for qualitative interviews. Including younger patients in interviews could have enriched the data, but reasons for not recruiting them remain unclear. Caring for younger patients with cancer is emotionally taxing (Burgers et al., 2022), potentially making it hard for HCPs to invite them to a study about what matters to them at the end of life. Due to the Covid pandemic, focus group interviews were conducted virtually. While these interviews provided rich data, understanding participant interactions during the discussions was challenging.

## Conclusion

When patients with cancer approach the end of life, their past experiences with healthcare services can lead to a lack of confidence in HCPs and the healthcare system. Patients in our study expressed feelings of exhaustion, loneliness, and abandonment. To address these needs, the development of various social interventions, along with the early integration of palliative care within municipalities, may enhance patients’ experiences. In focus group interviews, HCPs confirmed that transitioning to end-of-life care can be challenging for many patients. HCPs emphasized the importance of planning and predictability in end-of-life care. However, patients revealed a more ambiguous attitude; while they desired information and predictability, they were often reticent to discuss and plan for the final phase of life. Relational continuity and effective communication skills are crucial to improving end-of-life care. Close relationships emerged as significant to the patients in our study, although some described their relationships as poor. HCPs should be aware of this dynamic and support patients in navigating challenging family relationships. Finally, while many patients with cancer tend to be older, as observed in our study, the geriatric perspective appears to be lacking among HCPs.

Based on the results and limitations of our study, future research should explore the experiences and priorities of younger and middle-aged patients with cancer receiving home care at the end of life. Additionally, we recommend giving greater attention to geriatric perspectives in older patients with cancer.

## Supplementary Material

Appendix_Interview_guides.docx

## Data Availability

For ethical and data protection reasons, the authors are unable to make interview transcripts and internal administrative documents publicly available. The participants of this study did not consent to have their transcripts made publicly available. The transcripts are in Norwegian. Only quotes used in this article is translated to English.
