# Supplementary material for: What matters to patients with cancer receiving home care at the end of life? A qualitative study comparing patients’ and healthcare professionals’ views
Source: Int J Qual Stud Health Well-being. 2025 Jun 11;20(1):2517358. doi: 10.1080/17482631.2025.2517358 (PMC12160323; doi:10.1080/17482631.2025.2517358)
Supplement: Appendix_Interview_guides.docx [file ZQHW_A_2517358_SM8527.docx]

**Appendix**

**Interview Guide for Focus Group Interviews**

- How are healthcare services for patients with cancer in the end-of-life phase organized in the municipality?
- Based on your experience, what is most important for patients with cancer at the end of life? What do they prioritise? What has value for them? Do you have specific examples?
- What makes patients with cancer in the end-of-life phase have a good day? What brings joy? Do you have specific examples?
- What makes patients with cancer in the end-of-life phase have a bad day? What leads to sadness or depression? Do you have specific examples?
- Concerning the end of life, what comes to mind when you hear the term *quality of life*?
- What contributes to increasing the quality of life for patients with cancer at the end of life?
- What contributes to reducing the quality of life for patients with cancer at the end of life?
- How do the services currently offered meet patients’ need for security and good quality of life?
- Is there anything else that you would like to discuss regarding the quality of life of patients in the palliative phase?
- [Time permitting] What are some similarities and differences between patients with cancer and other types of patients?

**Interview Guide for Individual Interviews with Patients**

Tell me a little about yourself and your current situation regarding the course of the illness up to now.

**About life with cancer in the final stages of life:**

- We would like to know what a typical day is like for you. To help you answer, perhaps you can tell me about yesterday. How was it? What happened? Where were you? Who did you meet? How were you feeling? Was it a typical day for you now or not? If not, then what was different?
- [If not covered in the previous answer] You are at home, so can you manage on your own, or do you receive any help? If you receive help, then from whom? How much? What kinds of help?
- Have you given any thought to the future and where you wish to be? [Do you want to stay at home as long as possible? Die at home or somewhere else?]
- [If not covered in the previous answer] Who are you in contact with regarding the healthcare services that you receive? A general practitioner? Specialist health services? A palliative care team? Home care services? A cancer coordinator?

**About quality of life:**

- What is most important to you now? What do you prioritise?
- What has value for you now compared with earlier?
- Regarding the things that are most important to you, do they give your life quality? Or is quality of life something else?
- What do you think of when you hear the term *quality of life*, especially considering your current situation?
- What would improve your quality of life in your current situation? What brings you joy? [Alternatively: What makes you happy, and what makes life good? ]
- What would decrease your quality of life in your current situation? [Alternatively: What makes you sad/depressed? What makes your life difficult?]
- What factors currently affect your quality of life the most?
- Is there anything else that you would like to add regarding what we have discussed?
